# Cytokines and Angiogenesis in the Corpus Luteum

**DOI:** 10.1155/2013/420186

**Published:** 2013-06-11

**Authors:** António M. Galvão, Graça Ferreira-Dias, Dariusz J. Skarzynski

**Affiliations:** ^1^CIISA, Department of Morphology and Function, Faculty of Veterinary Medicine, Technical University of Lisbon, Avenida da Universidade Técnica, 1300-477 Lisboa, Portugal; ^2^Institute of Animal Reproduction and Food Research of PAS, Ulica Bydgoska 7, 10-243 Olsztyn, Poland

## Abstract

In adults, physiological angiogenesis is a rare event, with few exceptions as the vasculogenesis needed for tissue growth and function in female reproductive organs. Particularly in the corpus luteum (CL), regulation of angiogenic process seems to be tightly controlled by opposite actions resultant from the balance between pro- and antiangiogenic factors. It is the extremely rapid sequence of events that determines the dramatic changes on vascular and nonvascular structures, qualifying the CL as a great model for angiogenesis studies. Using the mare CL as a model, reports on locally produced cytokines, such as tumor necrosis factor **α** (TNF), interferon gamma (IFNG), or Fas ligand (FASL), pointed out their role on angiogenic activity modulation throughout the luteal phase. Thus, the main purpose of this review is to highlight the interaction between immune, endothelial, and luteal steroidogenic cells, regarding vascular dynamics/changes during establishment and regression of the equine CL.

## 1. Introduction

The angiogenic process plays an essential role during organogenesis and embryo development [1]. Angiogenesis itself can be classified as the process of new blood vessels formation from the preexisting vasculature. In adult tissues angiogenesis is very limited, and blood vessels remain quiescent until there is an angiogenic stimulus, such as hypoxia or wounding [2]. Besides the essential role of angiogenesis on wound healing, it also plays a major function in various diseases and tumorigenesis [3]. In contrast, after puberty, tissue growth and function of female reproductive organs (placenta, ovary, and corpus luteum) under physiologic conditions are extremely dependent on new blood vessels formation [4, 5].

Angiogenesis is a highly regulated process involving the balance between pro- and antiangiogenic factors. Locally, endothelial surrounding cells produce growth factors, cytokines, enzymes, receptors, adhesion molecules, and metabolic factors that regulate the angiogenic process. In this way, interaction between immune and endothelial cells should not be neglected [6]. It is well known that inflammatory cells, namely, macrophages, T lymphocytes, and monocytes, fully participate in the angiogenic process by secreting pro- and/or anti-inflammatory cytokines, which may control endothelial cells migration and activation, proliferation, survival, and apoptosis [7]. With particular regard to the ovary, it is well established that immune cells contribute for ovarian function regulation [8]. Moreover, immune cells present in the corpus luteum (CL) can be considered as a large pool of mobile cells that putatively modulate luteal establishment, maintenance, and regression. Overall, little is known about the complex cross-talk between immune and vascular systems in the CL. The plethora of intervenient cytokines and their often pleiotropic range of actions demand our attention to better understand luteal angiogenesis regulation. 

This review focuses on the modulation of angiogenesis by cytokines in the CL, addressing, in a chronological fashion, the events occurring from the follicle to the regressing CL. Special emphasis will be put on angiogenesis and cytokines action in the mare CL, since this research team has been gathering both descriptive and functional knowledge on the expression and role of cytokines like tumor necrosis factor alpha (TNF), interferon gamma (IFNG), Fas ligand (FASL), vascular endothelial growth factor A (VEGF), and nitric oxide (NO), among others, on equine luteal function. Due to the particular similarities between woman and mare (monovulatory species) on many aspects of ovarian function, mare CL is a valuable study model for understanding the regulatory pathways involved on the control of ovarian physiology [9]. Besides, most physiologic studies on woman ovarian function are based on the knowledge generated from abnormal tissue or granulosa cells collected from *in vitro *fertilization [10]. Therefore, their physiologic relevance is questionable [10], being the mare ovary a better study model.

## 2. Angiogenesis in the Corpus Luteum: A Chronological Sequence of Events from the Follicle to the Regressed Corpus Luteum

Different studies indicate that the CL is one of the most vascularized organs in the body [11, 12]. The CL undergoes extremely rapid cellular and vascular changes, only comparable with tumors [13]. The coordination of those biological processes is the outcome of a complex cross-talk between several factors. In the CL, as in other organs, angiogenesis seems to be tightly controlled by stimulating and inhibiting factors [14] that might regulate its vascularization and function [14, 15]. Development of the microvasculature during luteal establishment and formation is required for the delivery of adequate levels of hormones and lipoprotein bound cholesterol [16]. Quantitative reports in ruminants showed that in early luteal phase CL (early CL) more than 85% of proliferating cells are endothelial cells, while in mature CL more than 50% [12].

### 2.1. Starting from the Follicular Vasculature

During follicular growth, angiogenesis is determinant for preantral follicle development, follicle dominance, and preovulatory development [17]. Around 40% of proliferating cells in the theca are of endothelial origin [18]. Moreover, the blood clot formed during ovulation might stimulate cell migration. Indeed, platelets are a better stimulant for endothelial cells migration than granulosa cells themselves [19]. Examples of proangiogenic cytokines acting on this stage of the cycle include the cytokines fibroblast growth factor 2 (FGF2), VEGF, platelet-derived growth factor (PDGF) family, and angiopoietin (Ang). The VEGF has been described as the main proangiogenic factor, which is also produced by luteal cells [13, 14]. In fact, treatments with antiangiogenic compounds (VEGF trap) impaired follicular development [20]. Definitely, VEGF plays a central role, since its blockade abolished endothelial cell proliferation, luteal vascularization, and progesterone (P4) production in rat [21] and mouse CL [22]. 

In mares, dominant follicles show an increase in blood flow prior to deviation, when compared with subordinated follicles [23]. This follicular vascular bed provides the basis on which luteal vasculature will be formed [24]. It has been noticed that in the developing follicle granulosa and theca cells produce proangiogenic factors [25]. At the time of ovulation, the LH surge induces several important cellular and biochemical changes [26]. Specifically, breakdown of the basement membrane and immune-like responses are determinant for angiogenesis promotion [27]. Breakdown and reorganization of the blood vessel basement membrane involve a plethora of proteases, including matrix metalloproteinase (MMP) family, such as collagenases, gelatinases, and membrane-type MMP. Several MMPs (MMP9, MMP13, and MT-MMP1), which are primarily secreted by macrophages in the ovary of many species (reviewed by Wu et al. [28]), are upregulated by the LH surge [29]. It should be noticed that some of these MMPs also participate on the ovulatory process [30]. Thus, the physical block to vascularization of the granulosa layer is removed, and breaking down and spreading extracellular matrix (ECM) components take place, creating a more spacious environment facilitating motility and migration of endothelial cells and others. Another important consequence is the release of angiogenic factors sequestered in the basement membrane. Disintegrin and metalloproteinase with a thrombospondin (TSP) type 1 motif (ADAMTS) are proteases that appear to be critical for angiogenesis following ovulation [31]. The ADAMTS1 cleaves the matrix proteoglycans, expressed in the periovulatory follicle. Besides, ADAMTS1 is increased by gonadotropin stimulation [32], possibly mediated by hypoxia-induced factor 1*α* (HIF1A) pathway [33]. This might be important for endothelial cell invasion, since it is upregulated when these cells invade the collagen matrix, following VEGF and FGF2 stimulation [34].

Another important trigger for the increased blood flow is the HIF1A, whose expression is upregulated in the collapsed follicle of pigs [35], suggesting that this tissue is hypoxic. The relationship between LH, VEGF, FGF2, and HIF1A is still not clear for the period of follicular-luteal transition, but it is possible that VEGF raise following LH surge is mediated by HIF1A [36]. 

The cytokine TNF also presents an increased expression at the ovulation time [37], suggesting its participation on the ovulation process and incoming steps of luteal growth. Tumor necrosis factor *α* and its receptors presence was shown in the early CL of cow [38], pig [39], human [40], and horse (further discussed in detail) [41]. Regarding the TNF cellular action, the type of receptor involved should be considered. Indeed, TNF can induce both cell proliferation and death, depending on which receptor it binds to (TNFRI, the proapoptotic receptor; or TNFRII, the prosurvival receptor) [42, 43]. Reports in bovine CL demonstrated the TNF mediates endothelial cells proliferation [44–46]. Moreover, TNF participation in early CL vascularization should be considered alongside with the generation of NO. Studies by Okuda and coworkers evidenced that endothelial cells of bovine CL treated with TNF exhibited an increase in NO secretion [47], confirming the relevance of TNF/NO interaction on luteal angiogenesis in cows. The NO is considered a vasoactive substance, responsible for endothelial cells proliferation and VEGF secretion [48]. As shown for the mare, the NO donor (spermine NONOate) was able to stimulate angiogenic activity in early CL [49]. In parallel, the expression of NO generating enzyme, the endothelial NO synthase (eNOS), was increased in equine early CL [49], supporting NO role on luteal angiogenesis promotion.

Finally, as reviewed by Shirasuna and co-workers [50] polymorphonuclear leukocytes (PMNs) invade the CL soon after the ovulation. Neutrophils infiltration of bovine early CL (days 1–4) was correlated with a high concentration of interleukin 8 (IL-8, a neutrophil chemoattractant specific factor), suggesting that this cytokine also promotes angiogenesis in the CL [43, 44].

### 2.2. Luteal Endothelia Cell Migration and Proliferation

Endothelial cell migration involves its polarization towards an angiogenic stimulus, protrusion through filopodia-like structures, traction, and then retraction [51]. It is recognized that, in bovine CL, fibronectin forms a network of fibrils orientated along the axis of the capillary sprout [52], acting as a “prepatterned” guideline for endothelial cells migration. Fibronectin showed also a stimulatory effect on luteal-derived endothelial cell proliferation [53] and formation of endothelial cell networks *in vitro* [24]. Still considering the endothelial cell migration, it is believed that recently formed steroidogenic luteal cells can secrete the chemoattractants VEGF and FGF2, working on endothelial cells migration towards themselves [24]. 

The FGF2 also appears to be critical to endothelial network formation, since suppression of its receptor almost completely inhibited angiogenesis, by decreasing both the number of endothelial clusters and their size in the cow CL [24]. This occurred even in the presence of VEGF, emphasizing the importance of FGF2. Moreover, FGF2 expression increases during the initial stage of luteal formation, being a far more effective promoter of endothelial cell proliferation than VEGF [54]. It is suggested that both factors may have complementary rather than redundant actions on luteal angiogenesis [24]. 

Of particular interest for angiogenesis regulation in early CL is the modulation of FGF2 expression by prostaglandin (PG) F2*α*. As recently reported for early CL in cows (day 4), the so-called luteolytic PGF2*α* strongly increased FGF2 expression (mRNA and protein) [55]. From this standpoint, PGF2*α* would promote CL vascularization and support CL growth. In order to justify PGF2*α* putative effect on CL establishment, it was also suggested that PGF2*α* is able to interact with PGE_2_ receptors, when present in high concentrations [56]. Admittedly, the role of PGF2*α* on vascularization during CL growth awaits further research. However, the present evidence of PGF2*α* support on VEGF, FGF2, and P4 secretion in bovine CL confirms its participation on CL growth [55, 57].

### 2.3. A Mature Vascular System in the Corpus Luteum

Endothelial cells need structural support. Mural cells such as pericytes vascular smooth muscular cells ensure the shape and regulate blood flow through their contractile properties. The final step of angiogenesis is the vessel stabilization, achieved with the secretion of platelet-derived growth factor beta (PDGFB), which acts on a paracrine fashion on pericytes recruitment [58]. For many years the role of pericytes on angiogenesis was neglected. However, there is growing evidence of their importance on the promotion of angiogenesis initiation. During the ovulation time, pericytes are located at what appears to be the forefront of the endothelial migratory path [59], whilst in mature CL they are closely associated with endothelial cells. Furthermore, pericytes represent a large number of proliferating cells in the early ovine CL [59]. Firstly, pericytes act as guiding structures aiding the outgrowth of endothelial cells. They produce MMPs and may promote endothelial cell invasion, by destroying ECM. Then, pericytes are recruited for vessel stabilization [24]. Activation of these cells was associated with the platelet-derived growth factor (PDGF) system. Preovulatory treatment of mice with soluble ectodomain of PDGF receptor (PDGFR) prevented the recruitment of pericytes and reduced the staining of vascular area in CL [22], while the *in vitro* inhibition of PDGFR domain decreased the vascular network formation in bovine CL [54]. 

### 2.4. Luteolysis Demands Vascular Regression

A fundamental question concerning regression of the CL is whether regression of vasculature plays a role on functional and structural luteolysis. It was reported in sheep [60] and guinea pig [61] that apoptosis of endothelial cells presumably originated the occlusion of blood vessels with cellular debris. This could result in subsequent apoptosis of more endothelial cells followed by apoptosis of steroidogenic cells [62]. A pitfall determining the importance of endothelial cells apoptosis on luteolysis may be the fact that the temporal association between them diverges among species. The evidence in sheep and cow that PGF2*α* induces apoptosis of endothelial cells, resulting in a luteolytic cascade [63], is not that obvious in primates [64]. Nonetheless, death of vascular cells undoubtedly leads to a reduction in oxygen supply and nutrients to hormonal producing cells, perhaps contributing for their death.

The main luteolytic agent, the uterine PGF2*α*, has been associated with *in vivo* changes on vasculature. In fact, it has been proposed that the main consequence of PGF2*α* is the decrease in luteal blood flow [65]. However, following PGF2*α* administration, different responses are seen among species. In the cow, an acute increase on luteal blood flow was verified after 30 minutes to 2 h following administration of PGF2*α* [63]. A similar increase in blood flow at the beginning of luteolysis was not confirmed for the mare [66], whose luteal blood flow to the mid CL decreases some days before the decline in plasma P4 [67]. Several studies have related luteal blood flow changes seen in the cow with the potent vasorelaxant NO. The NO mediated raise in blood flow in cows accelerates neutrophils infiltration of the CL, mainly resulting in the production of various inflammatory cytokines production, such as IL-8, TNF, or INFG [68]. The IL1*β* was also related with luteolysis via NO synthesis promotion [69]. Additionally, these factors may be determinant for further luteal infiltration with immune cells (macrophages and T lymphocytes), supporting luteolytic cascade. Still regarding PGF2*α* effect on cellular changes during luteolysis, it was hypothesized that pericytes may serve as a regulator of tissue remodeling and integrity maintenance of large blood vessels, allowing normal luteolysis to occur [70]. Intriguingly, the pericytes, which are known to support angiogenesis [59], appear to participate in vascular regression during luteolysis.

The involvement of NO in equine CL regulation, specifically modulating changes in the vasculature, was recently described [49]. As previously mentioned (prior Section 2.1), eNOS protein was shown to be highly expressed in the mare early CL, when NO stimulated luteal tissue for angiogenic factors production and induced bovine aortic endothelial cells (BAEC) proliferation (used as a model to assess angiogenic factors production by luteal cells). Expression of eNOS was reduced in mid CL, and NO no longer increased BAEC mitogenic activity. In addition, participation of NO in vascular changes regulation throughout the luteal phase should be true also for the mare, since eNOS expression was increased once again in late luteal phase CL (late CL) [49]. Thus, NO is also involved in angioregulation during luteolysis.

The cytokines TNF and IFNG were shown to play a role in bovine luteal endothelial cells regulation [71]. Moreover, referred cytokines can interact with endothelin-1 (ET-1; mainly produced by endothelial cells) and PGF2*α*, inhibiting luteal steroidogenesis [72]. Two prior reports evidenced that TNF is cytotoxic for endothelial cells derived from bovine CL [73, 74]. Likewise, IFNG has been suggested as a locally secreted factor that may support TNF cytotoxic effect on bovine luteal endothelial cells [16]. Furthermore, cytokines TNF and IFNG can directly incite MCP-1 secretion and contribute for apoptosis of endothelial cells [61]. The present findings suggest that a cross-talk between immune and endothelial cells accounts for the increase in MCP-1 level and endothelial cell death, during PGF2*α*-induced luteal regression [75].

Changes in PGs and blood flow are considered necessary for local release of ET-1 and angiotensin II (ANGII), which further induce vasoconstriction and blood flow reduction [76]. Besides showing other biological functions, ET-1 is considered a potent vasoconstrictor, by acting on its receptor A [77]. Concerning the ANGII, it regulates several biological processes besides angiogenesis, including vascular tone and cellular growth. In cows, production of ANGII in the CL was associated with renin-angiotensin system [78]. Both ET-1 and ANGII can reduce luteal steroidogenesis and are considered vasoactive factors determinant for the luteolytic pathway and vascular regression [57]. 

Finally, vascular regression under the luteolytic context can be considered as a component of structural luteolysis. Generally, structural luteolysis implies strong ECM remodeling. As a result, MMPs participation is required once again but this time towards angioregression. It was demonstrated that, after PGF2*α* treatment, expression of MMPs (MMP1, MMP2, and MMP9) was increased, and this effect was potentiated by TNF [79]. Moreover, the same study showed that tissue inhibitor of metalloproteinase 1 (TIMP1, a specific inhibitor of matrix metalloproteinase) level was decreased during PGF2*α*-induced luteolysis, increasing the ratio of MMPs/TIMPs [79].

## 3. Vascular Regulation in the Equine Corpus Luteum

### 3.1. Angiogenic Function Characterization in the Equine CL

Since considerable differences are seen in the histology of the CL among species, the pattern of luteal vascularization should diverge. So far, few studies have described angiogenesis regulation in equine CL. Dynamic changes on vascular area in the mare CL were described throughout the luteal phase for the first time [15]. A marked increase in vascular area was seen in both early and mid CL, even though the vessel number was the highest in mid and late CL. The raise in DNA content seen from early to mid CL was associated not only with hyperplasia and luteal cell proliferation but also with endothelial cells proliferation [15]. Besides, the decrease in vascular area in the late CL might have been associated with the decrease in blood vessel lumen resultant from vessels contraction. This decrease in capillary diameter is considered determinant for blood flow fall and can initiate or accelerate luteal regression [80].

Among the various factors involved in luteal angiogenesis, VEGF appears to be the most important one for equine CL. It was evidenced that both VEGF mRNA transcription and protein expression peak in early and mid CL [81]. A direct temporal correlation with angiogenesis, blood vessels proliferation, and capillary density was established [81]. Besides, the presence in both follicular and luteal cells from equine ovary of VEGF, VEGF B, Ang1, Ang2, and the receptors VEGFR1, VEGFR2, and Tie2 was recently demonstrated [82]. A different staining was seen for VEGF, VEGFR2, and Ang2 in the periovulatory period (including the tertiary, the Graafian follicles, and early CL) [82]. These data showed their participation on equine luteal angiogenesis initiation. The Ang1 staining was mostly associated with arterioles, venules, arteries, and veins, compared with capillaries, suggesting a role on stabilization of this vasculature [83]. Regardless of the luteal stage, VEGFR1 was associated with mild expression intensity, and the complex VEGFB/VEGFR1 was not associated with proangiogenic events in the mare CL [82]. Furthermore, in the mature CL (mid CL) a more intense staining of proangiogenic studied factors could be observed specifically in the array of the vascular septa and in the CL periphery. These findings are in agreement with those from Al-zi'abi et al. [81]. In mid CL, capillary endothelial cells showed a less intense staining, mainly regarding VEGFR2 and Tie2, when compared with early CL. Also, luteal cells were characterized by a weaker immunolabeling for VEGFR2 in the mid CL [82].

### 3.2. Cytokines and Angiogenesis Regulation in the Equine CL

**Figure 1:**
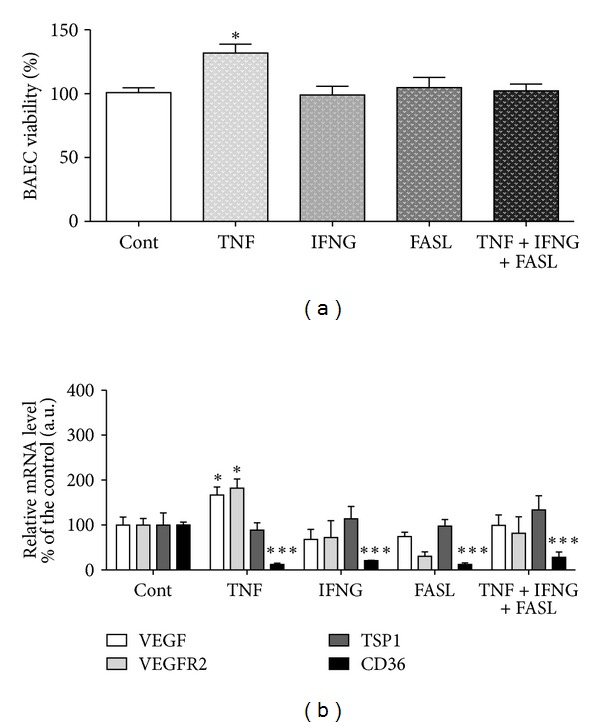
Figure adapted and modified from Galvão et al. [84]. (a) Bovine aortic endothelial cell (BAEC) proliferation rate, after incubation with conditioned media from luteal cells obtained from early CL (cytokines treatment for 24 h). (b) Relative quantification of VEGF, VEGFR2, TSP1, and CD36 mRNA transcription by real time PCR in early CL luteal cells (cytokines treatment for 24 h). Transcription normalized with the housekeeping gene—B2MG. Bars represent mean ± SEM. Asterisks indicate significant differences (*P < 0.05; ***P < 0.001), regarding the control values.

**Figure 2:**
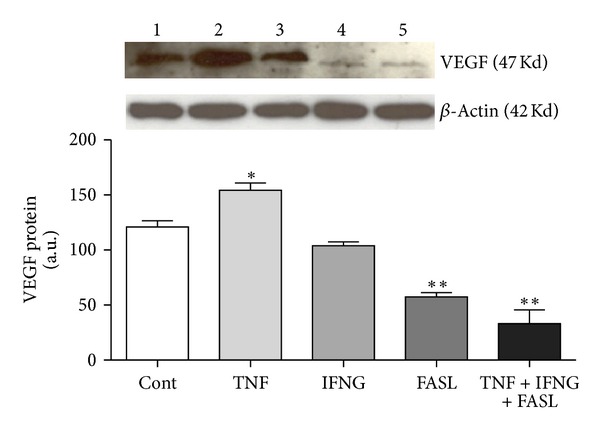
Figure adapted and modified from Galvão et al. [84]: VEGF protein expression in equine mid CL. Upper panels depict representative Western blot (*n* = 4). Lanes: (1) control; (2) TNF; (3) IFNG; (4) FASL; and (5) TNF + IFNG + FASL. Data normalized against *β*-actin density values. Bars represent mean ± SEM. Asterisks indicate significant differences (*P < 0.05; **P < 0.01).

*(i) Luteal Establishment. *Our team described the role of cytokines TNF, IFNG, and FASL on equine CL angiogenesis regulation. It is important to indicate that the cytokine TNF showed proangiogenic properties in early CL in the mare, after (i) increasing endothelial cells BAEC viability, (ii) increasing mRNA level of proangiogenic VEGF/VEGFR2 complex, and (iii) decreasing antiangiogenic CD36 (TSP1 receptor) [84] (Figure 1). Moreover, in mid CL treated cells, TNF increased VEGF protein expression [84] (Figure 2). By assessing BAEC viability, the ability of TNF to modulate angiogenesis by equine luteal cells was characterized (Figure 1(a)). In our study, VEGF and its receptor VEGFR2 mRNA levels were increased by TNF in the early CL (Figure 1(b)). Protein analysis also showed a stimulatory role of TNF on VEGF expression by mid CL cells (Figure 2). When the inhibitory effect of TNF on the mRNA level of the antiangiogenic receptor CD36 in early CL cells is taken into consideration, these findings suggest that TNF might participate in angiogenesis at the time of luteal formation in the mare. 

**Figure 3:**
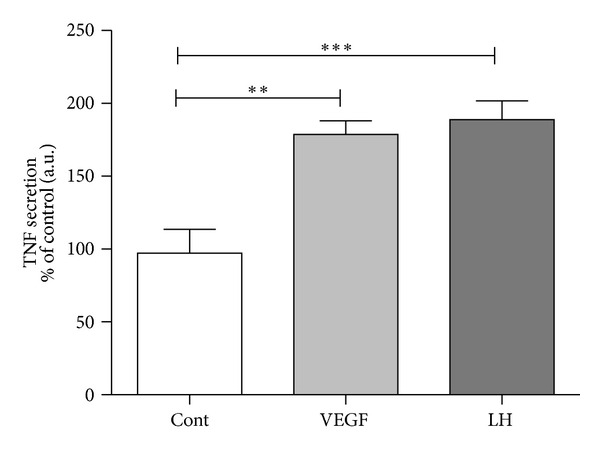
VEGF and LH action on TNF production by mid CL cells, after a 24 h stimulation. Stimulation dose for VEGF 50 ng/mL and for LH 10 ng/mL. Bars represent mean ± SEM. Asterisks indicate significant differences (**P < 0.01; ***P < 0.001). Luteal tissue and venous blood from jugular vein were collected after mortem at the local abattoir from randomly designated cyclic Lusitano mares. The luteal structures were classified in different luteal stages (early, mid, and late CL) as previously described [72, 82]. All methodologies for luteal cells isolation and culture, culture medium analysis by enzymatic immuno assay, and statistical analysis were recently described in detail [72].

**Figure 4:**
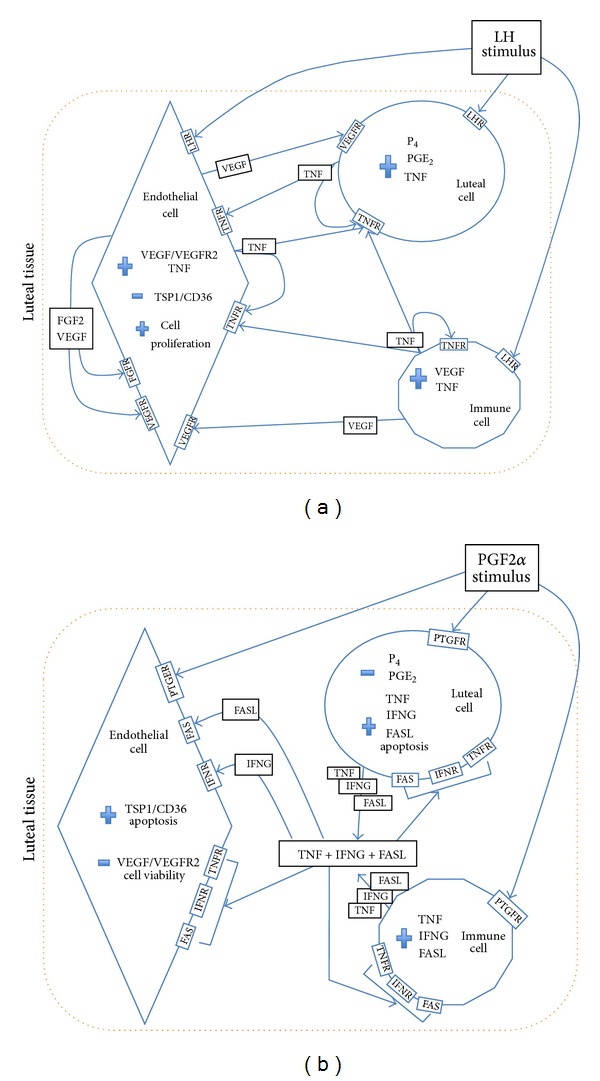
Schematic proposed interaction between endothelial, immune, and steroidogenic luteal cells in equine CL: (a) in early CL LH triggered luteotrophic loop between TNF, VEGF, and other factors during luteal growth; (b) in late CL PGF2*α* triggered luteolytic loop between cytokines FASL and cytokines synergic action TNF + IFNG + FASL towards angioregression and luteolytic cascade. Thick arrows indicate synergistic action of cytokines TNF + IFNG + FASL. The symbol + means increase in transcription/translation level; the symbol—means decrease in transcription/translation level. LH: luteotrophic hormone; LHR: LH receptor; PG: prostaglandin; PTGF: PGF2*α* receptor; P4: progesterone; TNF: tumor necrosis factor *α*; TNFR: TNF receptor; IFNG: interferon gamma; IFNR: IFNG receptor; FASL: Fas ligand; FAS: FASL receptor; FGF2: fibroblast growth factor 2; FGFR: FGF2 receptor; VEGF: vascular endothelial factor A; VEGFR2: VEGF receptor 2.

In spite of not being the only factor involved in endothelial cells promotion, VEGF is a crucial promoter of ovarian angiogenesis [20]. Thus, the interaction between TNF and VEGF may be determinant for luteal vasculature establishment. For instance, VEGF, which is secreted by macrophages in human ovary [28], was shown to be chemotactic for the same immune cell type, inducing neovascularization in mice [85]. Our group recently concluded that equine mid CL isolated cells treated with VEGF presented a raise in TNF secretion (Figure 3). This suggests the existence of a luteotrophic intraluteal loop, where TNF increases the VEGF production by equine luteal cells, and, in turn, VEGF synergically acts on TNF secretion. The notorious interaction between both immune and vascular systems here characterized is in agreement with previous findings in bovine CL [86]. Still considering the TNF/VEGF loop, the LH action should also be discussed. We demonstrated that mid CL LH treated cells presented an increase in TNF output, compared with control (Figure 3). As a matter of fact, LH is a major regulator of angiogenesis in several species [27], but its exact effect conducting luteal vascularization is not known. The *in vitro* stimulatory effect of LH on VEGF secretion by granulosa cells has been described in cows [86] and women [87]. In view of the present data, one may suggest that LH triggers TNF production during equine luteal angiogenesis promotion (Figure 4(a)), acting on different cellular departments, such as luteal cells, immune cells, or endothelial cells. As a result, TNF auto-, paracrine action (mainly on endothelial cells) stimulates VEGF production. The stimulus for vessels proliferation is then maintained with the VEGF action on luteal steroidogenic cells, via TNF transcription activation and transduction. Furthermore, TNF is a potent leukocyte chemoattractant factor, increasing this way the immune cells population in the developing CL (T cells, neutrophils, eosinophils, and macrophages are leukocytes present in the developing CL of several species—revised by Shirasuna et al. [50]). As mentioned before, VEGF is also chemotactic for macrophages [28], cooperating with TNF on CL immune cells infiltration.

*(ii) Luteal Regression. *In the mare, late CL conditioned media treated with PGF2*α* reduced mitogenic activity of BAEC [15]. In another study, after luteolytic PGF2*α* *in vivo* treatment, the expression of proangiogenic factors in the CL was reduced, and antiangiogenic factors production increased [88]. Also, after 12 h of induced luteal regression with PGF2*α*, on day 10 of the luteal phase, signs of swelling and apoptosis in equine luteal endothelial cells, as well as detachment from the blood vessels, were observed [88]. Active caspase-3 was also identified in large luteal cells and endothelial cells [74, 78]. In both steroidogenic and endothelial cells, the increase in caspase-3 expression was on day 14 of the luteal phase or 36 h after PGF2*α* administration [89]. Another important finding is the relationship between the onset of caspase-3 expression in endothelial cells on day 14 of luteal phase (or after luteolysis induction) and the decrease in mRNA and protein expression of VEGF in steroidogenic cells [81]. Nevertheless, in the mare there is no evidence that luteal endothelial cell death is the trigger for luteolysis, since death of endothelial cells is temporarily associated with death of steroidogenic cells.

**Figure 5:**
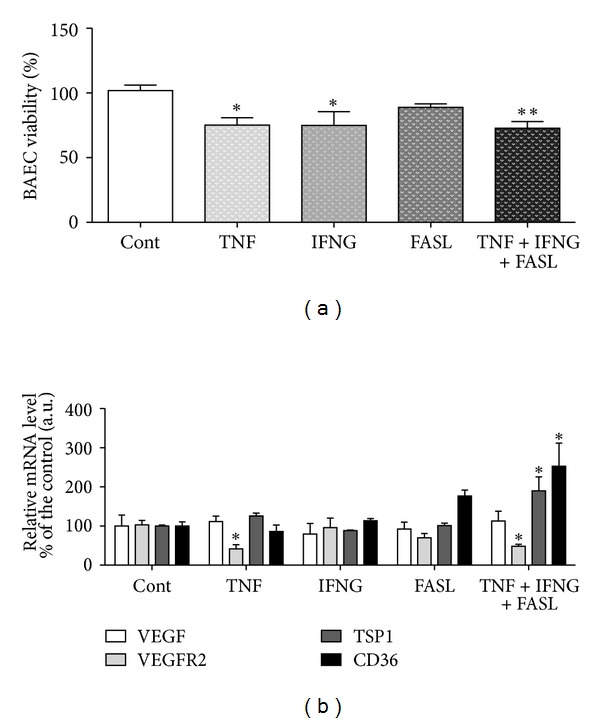
Figure adapted and modified from Galvão et al. [84]. (a) Bovine aortic endothelial cell (BAEC) proliferation rate, after incubation with conditioned media from luteal cells obtained from late CL (cytokines treatment for 24 h). (b) Relative quantification of VEGF, VEGFR2, TSP1, and CD36 mRNA transcription by real time PCR in late CL luteal cells (cytokines treatment for 24 h). Transcription normalized with the housekeeping gene—B2MG. Bars represent mean ± SEM. Asterisks indicate significant differences (*P < 0.05; ***P < 0.01), regarding the control values.

Regarding the immune-vascular interaction at the time of luteolysis, as previously reported in equine CL, macrophages population mainly increases in the late luteal phase [90]. Moreover, an influx of neutrophils was seen during spontaneous regression in hamsters CL [91]. Although neutrophils have been largely associated with phagocytosis, their participation in luteolysis has been also ascribed to cytokines secretion [91]. Hence, our recent work clearly stated the cytokines role on angiogenesis downregulation in equine CL [84]. In the late CL, a startling rise in antiangiogenic factors production after TNF treatment shows the stage specific role of this cytokine (Figure 5(a)) [84]. This definitely indicates TNF pleiotropy, suggesting that its modulation of angiogenic-signaling pathways depends on the local microenvironment and auto-, paracrine interactions with other factors.

As mentioned in Section 2.4, TNF deleterious action on bovine endothelial cells appears to be supported by IFNG [16]. In our work, the synergic action of these two cytokines on equine CL angiogenesis was not considered. Nevertheless, IFNG alone was able to decrease angiogenic activity in the late CL (Figure 5(a)), but no other changes were observed [84]. It should be also indicated that in the cow IFNG was associated with senescence and antiproliferative effects on specific luteal endothelial cell types [74].

Another interesting finding by this team was the demonstration of the negative effect played by FASL on VEGF protein expression (Figure 2). Our conclusions concerning FASL role on equine CL regulation show its importance besides the so-well characterized participation in structural luteolysis and apoptosis [92]. Initially, we have characterized FASL participation in luteal secretory impairment at the luteolysis time [93]. Since luteolysis is a dynamic process, we hypothesized that FASL could play a role in angiogenesis regulation. This cytokine is known to diminish angiogenesis in different organs [94]. Also in the mare, we have shown FASL specific downregulation of VEGF in the CL (Figure 2), which may trigger angioregression. 

We have also demonstrated that the cytokine association TNF + IFNG + FASL decreases VEGF protein expression in mid CL cells (Figure 2). In addition, cytokines association adequately restricted angiogenesis, after (i) increasing TSP1 and CD36 mRNA level, (ii) decreasing VGEFR2 mRNA level, and (iii) reducing BAEC proliferation in the late CL (Figure 5). When all the cytokines were tested together (TNF + IFNG + FASL), angiogenesis restriction was very effective in late CL cells (reduction of BAEC viability—Figure 5(a)). In late CL isolated cells, both TSP1 and CD36 mRNA levels were increased, while VEGFR2 was reduced (Figure 5(b)). Although no changes were seen in mRNA level, the same cytokine combination also reduced VEGF protein expression in mid CL cells (Figure 2).

The proteomic profile of these three cytokines (TNF, IFNG, and FASL) show that the increase in their expression [69, 82], and especially their synergic action, might be associated with functional luteolysis and consequently with angiogenesis downregulation (Figure 4(b)). Their combined action on angiogenesis regression was also demonstrated in the cow [16]. Thus, a temporal association between PGF2*α*, cytokines (TNF, IFNG, and FASL) increased expression, and VEGF reduced expression may be a major factor determining angioregression in the equine CL (Figure 4(b)).

## 4. Conclusion

A subnormal CL from the immediate previous estrous cycle will not prepare the uterus optimally for that gestation, ending in abortion. Despite the seriousness of this problem, the physiologic relevance of most studies on ovarian function in woman is questionable, since they are based on knowledge generated from abnormal tissue or granulosa cells collected from *in vitro* fertilization in women subjected to exogenous supraphysiological doses of gonadotropins [10]. Therefore, several investigators have proposed that tissues of female reproductive organs could serve as a model to study tissue growth/regression and angiogenesis in general [13]. Moreover, proximities on ovarian physiology between mares and women have been recently recognized [9]. Indeed, demonstrated similarities between those species in the dynamics of follicles during the interovulatory interval and during the ovulatory follicular wave endow the equine ovary with the best experimental model for studying ovarian function regulation [9]. Thus, we expect that produced data on ovarian angiogenesis modulation in the mare will significantly contribute for a better knowledge on the molecular mechanisms regulating luteal vascular growth and regression. With a short-term goal of a supportive application on assisted reproductive technologies, generated insights are likely to contribute for fertility improvement. Moreover, angiogenesis in general is better elucidated, with special impact on vascular diseases and tumorigenesis [3].

Many of the studies here cited represent a milestone in the depiction of this complex process of angiogenesis in the CL. It is now well established how crucial the interactions between different luteal cellular departments are. To be precise, immune cells, through their secreted cytokines, target gene expression and cell viability of both endothelial and steroidogenic luteal cells. This is a tight interaction where cytokines, through their auto-, paracrine actions, are seen as the main players. Hence, immune-vascular cross-talk was shown to be determinant for both luteal establishment and regression. Understanding the molecular regulation of these interactions will contribute for a better knowledge on angiogenesis regulation in general and luteal function in particular.
